# Electroacupuncture reduces calcium influx and inhibits endoplasmic reticulum stress by inhibiting the PKCδ-TRPA1 pathway to promote motor function recovery after spinal cord injury

**DOI:** 10.1093/burnst/tkaf066

**Published:** 2025-10-24

**Authors:** Yihui Zhang, Chenhe Liang, Zhiyang Huang, Yusi Hu, Yujun Mo, Shaoxia Ning, Ziyi Li, Yungang Wu, Jiaxin Zhang, Xiaokun Li, Zhouguang Wang

**Affiliations:** Department of Traditional Chinese Orthopaedics, the First Affiliated Hospital of Wenzhou Medical University, Nanbaixiang Street, Wenzhou City, Zhejiang Province 325035, China; Department of Traditional Chinese Orthopaedics, the First Affiliated Hospital of Wenzhou Medical University, Nanbaixiang Street, Wenzhou City, Zhejiang Province 325035, China; The First Affiliated Hospital of Wenzhou Medical University, Nanbaixiang Street, Wenzhou City, Zhejiang Province 325035, China; Wound Healing Center, the First Affiliated Hospital of Wenzhou Medical University, Nanbaixiang Street, Wenzhou City, Zhejiang Province 325035, China; National Key Laboratory of Macromolecular Drug Development and Manufacturing, School of Pharmaceutical Science, Wenzhou Medical University, Chashan Street, Wenzhou City, Zhejiang Province 325035, China; Alberta Institute, Wenzhou Medical University, Wenzhou, Chashan Street, Wenzhou City, Zhejiang Province 325035, China; Wound Healing Center, the First Affiliated Hospital of Wenzhou Medical University, Nanbaixiang Street, Wenzhou City, Zhejiang Province 325035, China; National Key Laboratory of Macromolecular Drug Development and Manufacturing, School of Pharmaceutical Science, Wenzhou Medical University, Chashan Street, Wenzhou City, Zhejiang Province 325035, China; Cixi Biomedical Research Institute, Wenzhou Medical University, No. 999, South Second Ring Road East, Hushan Street, Cixi City, Ningbo City, Zhejiang Province 315300, China; Cixi Biomedical Research Institute, Wenzhou Medical University, No. 999, South Second Ring Road East, Hushan Street, Cixi City, Ningbo City, Zhejiang Province 315300, China; Department of Traditional Chinese Orthopaedics, the First Affiliated Hospital of Wenzhou Medical University, Nanbaixiang Street, Wenzhou City, Zhejiang Province 325035, China; Department of Traditional Chinese Orthopaedics, the First Affiliated Hospital of Wenzhou Medical University, Nanbaixiang Street, Wenzhou City, Zhejiang Province 325035, China; National Key Laboratory of Macromolecular Drug Development and Manufacturing, School of Pharmaceutical Science, Wenzhou Medical University, Chashan Street, Wenzhou City, Zhejiang Province 325035, China; Department of Traditional Chinese Orthopaedics, the First Affiliated Hospital of Wenzhou Medical University, Nanbaixiang Street, Wenzhou City, Zhejiang Province 325035, China; Wound Healing Center, the First Affiliated Hospital of Wenzhou Medical University, Nanbaixiang Street, Wenzhou City, Zhejiang Province 325035, China; National Key Laboratory of Macromolecular Drug Development and Manufacturing, School of Pharmaceutical Science, Wenzhou Medical University, Chashan Street, Wenzhou City, Zhejiang Province 325035, China

**Keywords:** Spinal cord injury, Electroacupuncture, PKCδ/TRPA1, Endoplasmic reticulum stress, Neurogenesis

## Abstract

**Background:**

Following spinal cord injury (SCI), mechanical trauma and an inflammatory microenvironment activate the PKCδ-TRPA1 pathway, resulting in calcium overload within neurons and subsequently inducing endoplasmic reticulum (ER) stress–mediated neuronal apoptosis. The mechanisms and therapeutic potential of electroacupuncture (EA) in the treatment of SCI have yet to be fully elucidated. This study aimed to explore the causes of neuronal Ca^2+^ overload post-SCI and to investigate the neuroprotective and regenerative mechanisms of EA in an SCI mouse model.

**Methods:**

C57BL6 mice were randomly divided into a Sham group, an SCI group, and an SCI + EA group. The Basso Mouse Scale, motor-evoked potential, and movement videos of the mice were captured, and DeepLabCut was used to analyze the recovery of motor function. Western blotting was used to detect the protein levels of related indicators. Immunofluorescence staining was used to analyse the cellular localization and fluorescence intensity of each indicator. Hematoxylin and eosin staining and Nissl staining were used for histological evaluation. EA treatment resulted in higher BMS scores; increased ankle, knee, and hip mobility; and improved hindlimb support in the EA group.

**Results:**

PKCδ and TRPA1 protein expression was upregulated after SCI, and neuronal calcium ion overload occurred, leading to neuronal ER stress–induced apoptosis. After EA treatment, inflammatory microenvironment-related indicators were downregulated, which inhibited the activation of calcium channels by PKCδ-TRPA1, reduced ER stress–induced apoptosis caused by calcium overload in neurons, and protected neurons from secondary injury. EA increases the expression of neurotrophic factors and promotes nerve regeneration, and we found that EA treatment promotes axonal elongation by stabilizing microtubules.

**Conclusions:**

According to our findings, (i) mechanical injury and the inflammatory microenvironment after SCI activate PKCδ-TRPA1, which is important for neuronal ER stress and apoptosis caused by neuronal calcium overload. (ii) EA treatment reduces the expression of PKCδ-TRPA1 as well as the ER stress and apoptosis caused by neuronal calcium overload. (iii) EA promoted neurogenesis and axonal regeneration by promoting the secretion of neurotrophic factors and promoted axon elongation by stabilizing microtubules, thus promoting the recovery of motor function in SCI mice.

## Graphical Abstract

**Graphical Abstract:**
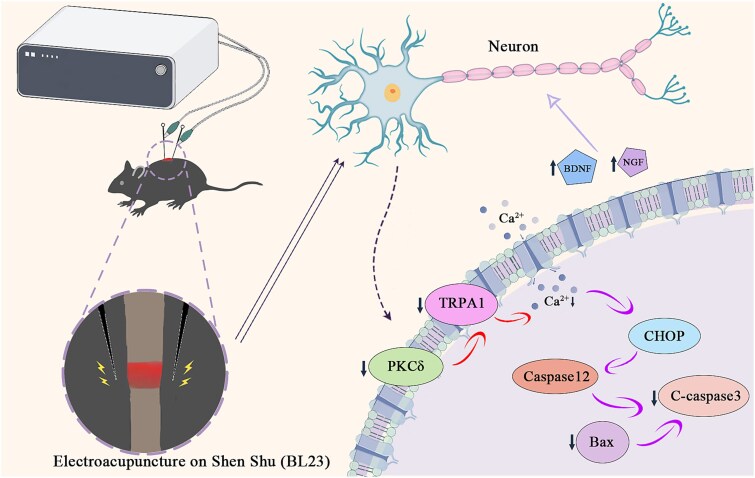

## Highlights

- The activation of PKCδ-TRPA1 induced by mechanical injury and the inflammatory microenvironment after SCI is a significant cause of neuronal calcium overload, which subsequently leads to ER stress and apoptosis.
- EA stimulation at the Shen Shu points can reduce the expression of PKCδ-TRPA1 and alleviate ER stress and apoptosis induced by neuronal calcium overload.
- Stimulation of the Shen Shu points by EA promoted neurogenesis and axon elongation by enhancing the secretion of neurotrophic factors and stabilizing microtubules, thereby facilitating the recovery of motor function in SCI mice.

## Background

Traumatic spinal cord injury (SCI) is a condition with a high incidence rate that has been progressively increasing in recent years, often resulting in the loss of motor and sensory function in the lower limbs [1, 2]. Following SCI, the secondary injury process involving a significant amount of neuronal death is particularly critical, with an imbalance in calcium homeostasis being among the core mechanisms [3]. In the initial phase following injury, neurons are under severe stress, with membrane potential and ion channel dysfunction, resulting in excessive calcium influx [4]. Abnormal calcium influx not only directly damages mitochondria but also significantly increases the endoplasmic reticulum burden and induces an endoplasmic reticulum (ER) stress response [5, 6]. Continuous ER stress activates caspase family proteins, thereby mediating neuronal apoptosis and axonal disruption and further aggravating neurological deficits [7]. Thus, maintaining calcium homeostasis and mitigating ER stress play crucial roles in inhibiting the secondary pathological processes of SCI and promoting the survival and functional recovery of neurons.

TRPA1 is a member of the transient receptor potential (TRP) channel family and functions as a nonselective cation channel [8]. This channel can be activated by various stimuli, including mechanical vibration, temperature changes, and inflammatory mediators such as reactive oxygen species and pain-related factors [9, 10]. TRPA1 plays a critical role in pain transmission and the regulation of neuronal excitability. The channel is opened upon TRPA1 activation, resulting in a large amount of Ca^2+^ influx [11]. The magnitude of this influx can exceed physiological homeostasis levels by several times, which becomes one of the main sources of intracellular calcium overload. This disrupts the calcium balance in the ER and induces sustained ER stress as well as CHOP/caspase-12-mediated apoptosis in neurons [12, 13].

During the local inflammatory response following SCI, Tumor necrosis factor α (TNF-α) and Interleukin-1β‌ (IL-1β) levels are significantly elevated. This elevation can lead to the activation of various protein kinase C (PKC) isoforms [14, 15]. PKC activation simultaneously phosphorylates multiple sites in the C-terminal region of TRPA1, thereby significantly increasing the sensitivity of the channel to stimulation and amplifying the influx of Ca^2+^ [16, 17]. PKCδ, as a key site among PKC subunits affected by inflammation [18, 19], may play an important role in the downstream Ca^2+^ influx triggered by TRPA1 channel sensitivity. This establishes a self-reinforcing feedback loop in which Ca^2+^ overload exacerbates ER stress, which subsequently upregulates TRPA1 expression and sensitivity, thereby further increasing Ca^2+^ influx [20]. It establishes a detrimental feedback loop characterized by persistent calcium overload and ER stress, thereby impairing neuronal survival and axon regeneration. Modulating this pathway represents a promising strategy for mitigating secondary injury in SCI.

Acupuncture is an ancient Chinese medical technique with a rich historical background and has been utilized in the treatment of various nervous system disorders, including Parkinson’s disease and epilepsy [21]. Additionally, acupuncture serves as an adjuvant therapy following SCI, primarily by alleviating pain, enhancing neuroplasticity, and modulating immune responses, thereby facilitating the recovery of motor and sensory functions in patients with SCI [22]. Electroacupuncture (EA) is an integrative therapeutic modality that combines traditional manual acupuncture with pulsed electrical stimulation [23], and its neuroprotective effect against central nervous system injury has been recognized [24, 25]. Clinical trials have demonstrated that compared with traditional rehabilitation therapies, EA administered at specific governor vessel points, such as GV6 and GV9, increases lower limb motor function scores in SCI patients by 15%–20% [26]. In preclinical studies, EA at a frequency of 4–20 Hz reduced SCI-induced glial scar formation and enhanced axon regeneration through BDNF/TrkB signalling. In another study, EA inhibited the protective effect of neuroinflammation against ischaemic brain injury by inhibiting TRPV4 and M1 polarization of microglia/macrophages [27]. TRPA1 is activated by the physical and chemical stimuli associated with SCI. In the secondary phase of SCI, the PKCδ-TRPA1 pathway remains activated continuously in response to neuroinflammation and reactive oxygen species, leading to a significant increase in intracellular calcium levels. In this study, we examined the potential of EA stimulation of Shen Shu points to mitigate calcium overload and ER stress through inhibition of the PKCδ-TRPA1 axis.

The therapeutic efficacy of EA is evident not only in the stabilization of the microenvironment during the secondary phase but also in the influence of low-frequency electrical stimulation on neurotrophic factors. Research has demonstrated that low-frequency electrical stimulation can promote the secretion of nerve growth factor (NGF) and brain-derived neurotrophic factor (BDNF), thereby increasing the survival [28, 29], proliferation, and differentiation of neurons, ultimately accelerating neural repair.

Currently, the mechanisms underlying the effects of EA in SCI remain poorly understood. Therefore, revealing a new mechanism through which EA affects SCI recovery can provide a theoretical basis for the treatment of nerve trauma. In our study, we employed EA to stimulate the Shen Shu acupoints to evaluate whether inhibition of the PKCδ-TRPA1 axis following treatment could mitigate neuronal calcium overload and ER stress, thereby reducing neuronal apoptosis during the secondary stage. Motor function recovery in response to EA stimulation at the Shen Shu point was evaluated using behavioural analyses. In conclusion, our research elucidated a novel mechanism through which EA facilitates SCI recovery and established a theoretical foundation for the role of the PKCδ-TRPA1 signalling pathway as a potential therapeutic target for SCI treatment.

## Methods

### Reagents and antibodies

Primary antibodies against PKCδ (14188-1-AP), TRPA1 (19124-1-AP), GRP78 (11587-1-AP), BAX (50599-2-Ig), Bcl-2 (60178-1-Ig), CHOP (15204-1-AP), CD206 (18704-1-AP), and Brdu (66241-1-Ig) were obtained from Proteintech. Primary antibodies against NGF (DF6061) and NGFR (DF6821) were obtained from Affinity. Primary antibodies against CD86 (sc-28 347) were obtained from Santa Cruz. Primary antibodies against Arg-1 (93668S) and C-caspase3 (9661S) were obtained from Cell Signaling Technology. Primary antibodies against Caspase12 (ab235180), NeuN (ab104224), GFAP (ab7260), MAP-2 (ab5392), NF200 (ab4680), and alpha-tubulin (ab179484) were obtained from Abcam. Primary antibodies against alpha-tubulin (tyrosinated, SAB4200776) were obtained from Millipore. Primary antibodies against DCX (PA5-142703) were obtained from Thermo. Primary antibodies against beta-actin were obtained from Zen-bio, and anti-rabbit (ab6721) and anti-mouse (ab6728) secondary antibodies conjugated with horseradish peroxidase (HRP) were obtained from Abcam. The secondary antibodies anti-mouse 488 (ab150109), anti-mouse 555 (ab150110), anti-rabbit 488 (ab150077), anti-rabbit 555 (ab150062), anti-rabbit 647 (ab150063), anti-chicken 488 (ab150169), anti-chicken 647 (ab150175), and anti-goat488 (ab150129) were obtained from Abcam. A haematoxylin and eosin (H&E) staining kit (G1430) and Nissl staining solution (G1120) were obtained from Solarbio. The TRPA1 agonist JT010 (HY-111132) was obtained from MCE.

### Model of spinal cord injury

C57BL/6 female mice (weighing 20–22 g; aged 8–10 weeks) were supplied by the Animal Center of Hangzhou Medical College. These mice were randomly divided into three groups: the Sham group (*n =* 30), the SCI group (*n =* 30), and the EA group (*n =* 30). All experimental procedures were approved by the Animal Research Committee of The First Affiliated Hospital of Wenzhou Medical University (reference number WYYY-AEC-YS-2023-0490). Such experiments were conducted in strict compliance with the National Institutes of Health guidelines for the care and use of laboratory animals. Prior to the operation, each mouse was anaesthetized via intraperitoneal injection of a 1% sodium pentobarbital solution at a dose of 50 mg/kg. Standard laminectomy was performed at the T9–T10 vertebral levels to expose the intact spinal cord. T9 spinal cord contusion using a Rewald spinal cord percussor: severe spinal cord injury (parameters: 1.5 m/s, 0.6 mm). After SCI, cefazolin sodium (0.9%) was administered intraperitoneally twice daily, and the rats were allowed to urinate artificially three times daily until the bladder reflex recovered. The mice in the sham group underwent anaesthesia and laminectomy without spinal cord injury.

### Electroacupuncture treatment

On the first day after the operation, disposable sterilized stainless-steel needles were used. The needle was inserted into the Shen Shu point (BL 23) (located 5 mm lateral to the spinous process of the second lumbar vertebra) at a depth of 3 mm. An electroacupuncture instrument (SDZ-IV; Hua Tuo, China) was used. The current range was set at 1.0 mA, the control frequency was 4/10 Hz, and the time was 15 minutes. The treatment was continued once a day for 14 days.

### Electrophysiology

After the mice were anaesthetized with 1% sodium pentobarbital, a stimulating needle electrode (DSN1620; Medtronic, USA) was placed at the upper end of the injured spinal cord, a recording electrode was inserted into the tibialis anterior muscle, and a ground electrode was placed under the skin of the hip. An electrical stimulator (Keypoint, Medtronic, USA) was used to stimulate the spinal cord with 10 mA, 0.1 ms, and 1 Hz electrical stimulation pulses, with an interval of 15 s. The amplitude of evoked potentials after each stimulation was recorded [30, 31].

### Motor function assessment

The Basso Mouse Scale (BMS) was used to evaluate the recovery of hind limb motor function in SCI mice at 1, 3, 7, 14, 21, and 30 days after intervention. The mouse was placed on a straight track, the hind foot of the mouse was marked with red pigment, the mouse was allowed to move freely forwards, and the hind limb motor function was evaluated by red footprints. The mice were subsequently placed on fixed tracks for more detailed kinematic analysis. A high-frame-rate video was used to record the linear movement process of the mice on the track. The iliac joint, hip joint, knee joint, ankle joint, and toe joint of the mice were marked by DeepLabCut [32, 33], and the movement trajectory of the mice was analysed. MATLAB was used to construct the mouse hindlimb movement stick diagrams, as well as the angle diagrams of the hip joints, knee joints, and ankle joints.

### BrdU administration

After BrdU (HY-15910, MCE, 0.5 g/L) was added to the drinking water, the proliferating cells were labelled *in vivo*. Afterwards, the samples were sectioned, the DNA was denatured with DNAse I, and BrdU antibody staining was performed.

### Corticospinal system (CST) tracing experiment

For the CST tracing experiment, AAV2/9-hSyn-hChR2(H134R)-mCherry-WPRE (Obio Biotechnology) was injected into the right sensorimotor cortex of each mouse (at a titre of 2.08 × 10^13^). The injection coordinates were 1, 0.5, 0, −0.5, −1 and − 1.5 mm caudally; 1.3 mm laterally from the anterior fontanelle; and 0.6 mm ventrally from the brain surface (6 injection points on the right side, with 100 nl injected each time) [34].

### Western blot analysis

Spinal cord tissue was excised from the central region of the SCI site and immediately stored at −80°C for Western blot analysis. The tissues were homogenized in RIPA lysis buffer supplemented with Phenylmethylsulfonyl fluoride‌ (PMSF) and phosphatase inhibitor. Following homogenization, the samples were centrifuged at 12 000 rpm for 20 min at 4°C, after which the resulting supernatant was collected. The protein concentration of the supernatant was determined using Coomassie Brilliant blue. A consistent amount of protein (40 μg per lane) was loaded for electrophoretic separation and subsequently electrotransferred onto a Polyvinylidene difluoride Membrane (PVDF) membrane. After the membrane was blocked for 15 min, it was incubated with primary antibodies overnight at 4°C. On the following day, after three washes with TBST, the membrane was incubated with an HRP-conjugated secondary antibody for 2 h at room temperature. The signals were detected using a ChemiDoc XRS+ imaging system (Bio-Rad). Band densities were quantified using ImageJ software.

### Immunofluorescence staining

The harvested spinal cord was fixed in 4% paraformaldehyde for 2 days and embedded in paraffin. The sections were deparaffinized in xylene, rehydrated with gradient ethanol, subjected to antigen retrieval using 0.01% sodium citrate buffer, and blocked with 1% bovine serum protein (BSA) for 30 min. The sections were incubated overnight at 4°C with the appropriate primary antibodies. On the following day, after three washes with Phosphate-Buffered Saline with Tween 20 (PBST), the slides were incubated for 1 h at room temperature with the corresponding secondary antibodies, followed by 4′,6-Diamidino-2-phenylindole (DAPI) sealing. Images were acquired using a laser confocal microscope and analysed using ImageJ software. Statistical graphs were generated using Prism 7 (GraphPad Software, San Diego, CA).

### Haematoxylin and eosin staining

The tissues from each group were embedded in paraffin and sectioned at a thickness of ~5 μm. H&E (Solarbio, G1120, experimental procedures as described in the instructions) was used for pathological examination. Microscopic observations and image acquisition were performed using an optical microscope.

### Nissl staining

After deparaffinization and hydration, the paraffin sections were stained with tar violet dye at 56°C for 45 min. The sections were subsequently washed with distilled water, and Nissl differentiation solution was used for 1 min, followed by dehydration, transparency, and sealing. Microscopic observations and image acquisition were performed using an optical microscope.

### Statistical analysis

The BMS is used to assess the motor function of the hind limbs in mice and is an ordered set of repeated measurements. It is analysed using a generalized linear mixed model, which employs a cumulative logit link function and includes individual ID as a random effect to account for the correlation within individuals. For continuous variables with a normal distribution, one-way analysis of variance (ANOVA) was used for single-factor comparisons. When both treatment group and time were factors, two-way repeated-measures ANOVA was employed, followed by Tukey’s post hoc multiple comparisons test. Statistical analysis was performed using Excel and GraphPad Prism 7. The data are displayed as the mean ± standard deviation (SD). *P* < 0.05 indicated a significant difference.

## Results

### Electroacupuncture promotes the recovery of motor function in mice

**Figure 1:**
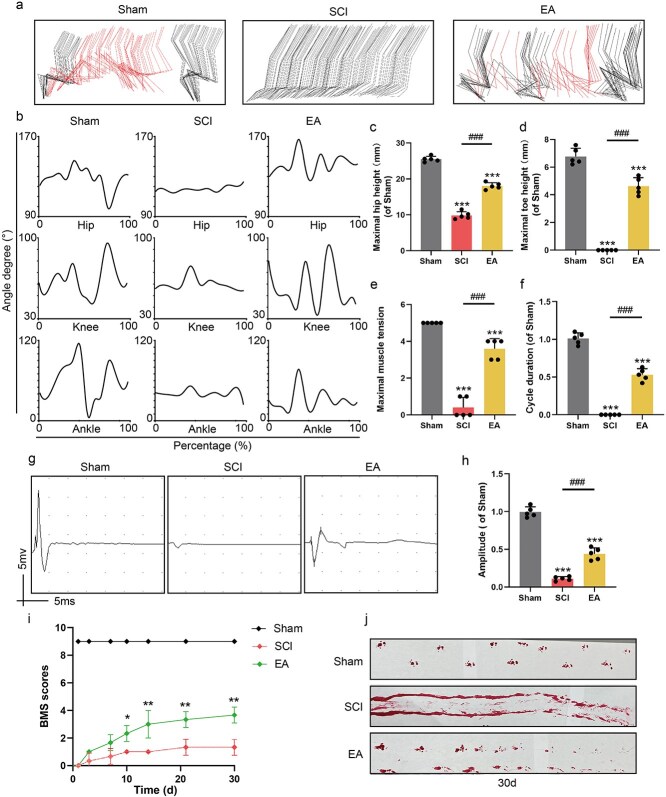
EA can promote the recovery of motor function in SCI mice. (**a**) Representative colored stick plot of hindlimb movements of mice exercising in the Sham, SCI, EA group. (**b**) Amplitude statistics of ankle, knee, and hip joints of mice in each group. (**c**–**f**) Quantitative analysis of maximum hip height, maximum toe height, maximum muscle tone, and movement cycle distance in different treated mice. (**g**, **h**) Electromyography and analysis of each group. (**i**) BMS score in the Sham, SCI, EA group. (**j**) Footprints in each group. ^***^  *represents P* < 0.001. ^###^ represents the SCI group *vs* EA group, respectively, *P* <0.001. The data are represented as mean ± SD (*n =* 5). *EA* electroacupuncture, *SCI* spinal cord injury.

In the detailed motion video analysis, we employed DeepLabCut to annotate the mouse’s iliac joint, hip keypoint, knee joint, ankle joint, and toe joints to examine the movement trajectories of the mouse. We observed that the hindlimb movement of SCI mice was characterized by a dragging motion during forwards progression. However, in the EA group, obvious elevation of the knee joint, ankle joint, and toe joint was observed, demonstrating that the ability of the lower limbs of the mice was restored and that the support performance of the hind limbs to the body was significantly improved (Figure 1a). Moreover, the amplitudes of the ankle, knee, and hip joints in the EA group increased (Figure 1b). The mice in the EA group exhibited significantly greater hip height (Figure 1c), greater height of the hind limbs (Figure 1d), and greater muscle tone (Figure 1e), and achieved the longest single movement cycle (Figure 1f). Subsequently, electromyography was used to determine whether EA could increase Electromyography (EMG) signalling upstream of the injury area to the gastrocnemius muscle. The SCI group was only capable of transmitting weak electrical signals, whereas the EA group exhibited markedly enhanced transmission of electrical signals, characterized by a significant increase in amplitude (Figure 1g and h). The scores of the BMS were significantly greater in the EA group than in the control group at 1, 3, 7, 14, 21, and 30 days (Figure 1i). In the foot imprint analysis, the hind limbs of the SCI group exhibited dragging, whereas those in the EA group showed clear footprints with distinct movement cycles (Figure 1j). In conclusion, EA significantly enhances motor function recovery in mice.

### Electroacupuncture reduces the inflammatory microenvironment in mice after spinal cord injury

**Figure. 2:**
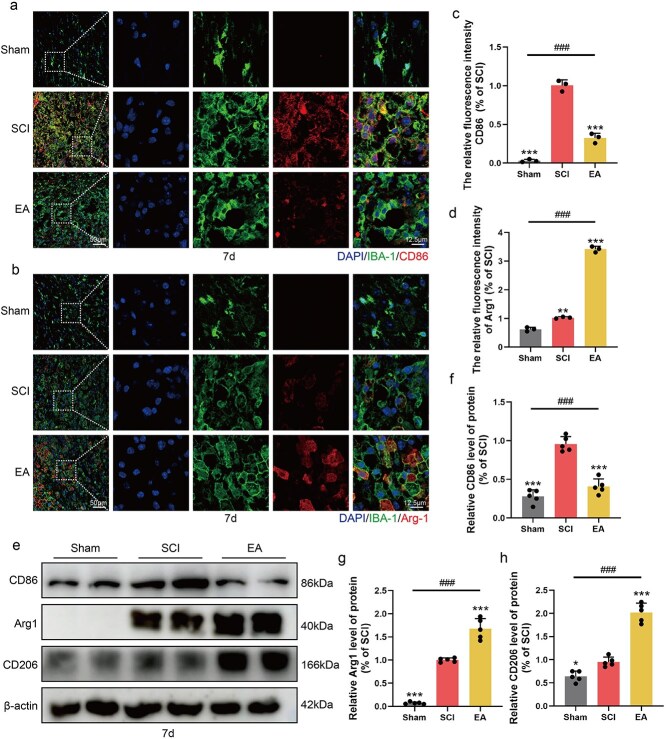
EA treatment can improve the inflammatory microenvironment in SCI mice. (**a**) Immunofluorescence staining showed the expression of DAPI (blue), IBA-1 (green), and CD86 (red) in the Sham, SCI, EA group. Scale bar: 50 μm; 12.5 μm.(**b**) Immunofluorescence staining showed the expression of DAPI (blue), IBA-1 (green), and Arg-1 (red) in all groups 7 days post-SCI. Scale bar: 50 μm; 12.5 μm. (**c**, **d**) Statistical analysis of fluorescence intensity of CD86 and Arg-1 in each group, and the data are represented as mean ± SD (*n =* 3). (**e**) Western blotting showing the expression of CD86, Arg-1, CD206 in each group. (**f**–**h**) Quantitative analysis of protein expression in each group and the data are represented as mean ± SD (*n =* 5). ^*^represents *P* < 0.05, ^***^represents *P* < 0.001. *###* represents the sham group *vs* the EA group, *P* < 0.001. n.s. represents no statistical significance. *EA* electroacupuncture, *SCI* spinal cord injury.

The inflammatory microenvironment following SCI exerts significant destructive effects during the secondary injury phase, contributing substantially to widespread apoptosis. EA can modulate the balance of inflammatory factors within this microenvironment via multiple signalling pathways. Immunofluorescence staining revealed that a large number of CD86 (M1-type microglia)- and IBA-1-positive microglia were colocalized after SCI, indicating that the injured area was in a state of high inflammation. After EA treatment, the CD86 fluorescence intensity decreased in the injured area (Figure 2a and c). In addition, the fluorescence intensity of Arg-1 was significantly increased after EA treatment, and the colocalization of high levels of Arg-1 (M2 microglia) and IBA-1 increased, indicating that EA treatment promoted the conversion of microglia from the M1 phenotype to the M2 phenotype (Figure 2b and d). In conjunction with the Western blot results, these findings revealed that EA treatment led to a reduction in CD86 expression but upregulated the expression of Arg-1 and CD206 following SCI (Figure 2e–h). In conclusion, our findings indicate that EA mitigates the inflammatory microenvironment and facilitates the polarization shift from M1 microglia to M2 microglia in mice following SCI.

### Electroacupuncture treatment reduces neuronal endoplasmic reticulum stress through the PKCδ-TRPA1 pathway

**Figure 3:**
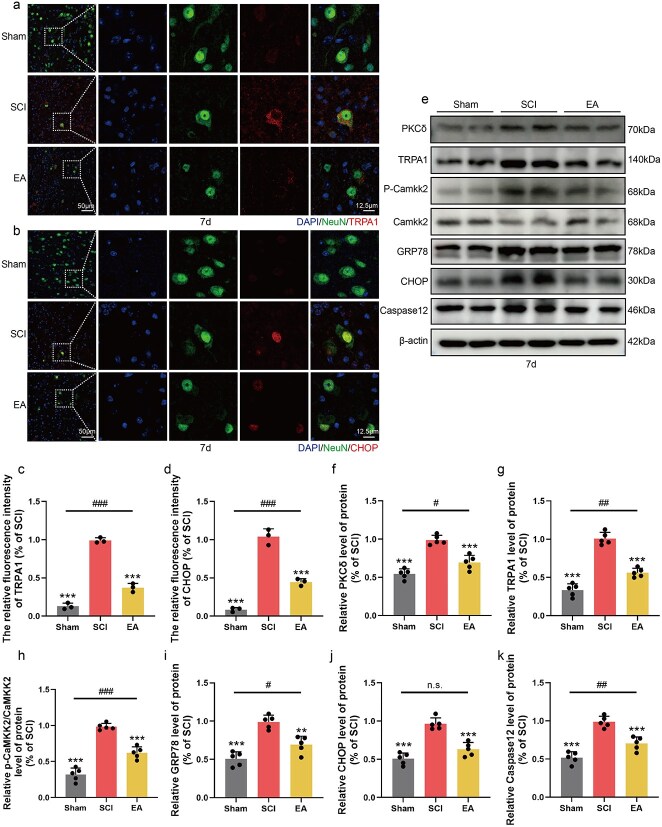
EA treatment mitigates neuronal Ca^2+^ overload and ER stress following SCI through the PKCδ-TRPA1 pathway. (**a**) Immunofluorescence staining showed the expression of DAPI (blue), NeuN (green), and TRPA1 (red) in all groups 7 days post-SCI. Scale bar: 50 μm; 12.5 μm. (**b**) Immunofluorescence staining showed the expression of DAPI (blue), NeuN (green), and CHOP (red) in all groups 7 days post-SCI. Scale bar: 50 μm; 12.5 μm. (**c**, **d**) Statistical analysis of fluorescence intensity of CHOP and TRPA1 in each group, and the data are represented as mean ± SD (*n =* 3). (**e**) Western blotting showing the expression of PKCδ, TRPA1, P-Camkk2/Camkk2, GRP78, CHOP, and Caspase12 in each group. (**f**–**k**) Quantitative analysis of protein expression in each group and the data are represented as mean ± SD (*n =* 5). ^**^represents *P* < 0.01, ^***^represents *P* < 0.001. ^#^, ^##^, ^###^ represent sham group *vs* EA group, respectively, *P* < 0.05, *P* < 0.01, *P* < 0.001. n.s. represents no statistical significance. *EA* electroacupuncture, *SCI* spinal cord injury.

To verify the therapeutic effect of EA, we first investigated the regulatory effect of calcium ions on the PKCδ-TRPA1 pathway after SCI by western blotting. PKCδ and TRPA1 protein levels were significantly increased after SCI, and Camkk2 phosphorylation increased, indicating that the microenvironment failed to regulate calcium channels after injury to the PKCδ-TRPA1 pathway and that abnormal opening of calcium channels led to a significant increase in the intracellular calcium concentration. PKCδ-TRPA1 protein levels were downregulated following EA treatment, potentially because of the mitigation of the inflammatory microenvironment and decreased ion channel sensitivity. Additionally, Camkk2 phosphorylation is typically induced by an increase in the intracellular calcium ion concentration. We found that in addition to downregulation of the PKCδ-TRPA1 pathway, P-Camkk2 expression decreased after EA treatment (Figure 3e–h). In the tissue immunofluorescence experiment, costaining of the neuronal markers NeuN and TRPA1 revealed that the SCI group exhibited the most prominent colocalization of NeuN and TRPA1. This colocalization was significantly reduced following EA treatment (Figure 3a and c).

Disruption of calcium channels mediated by PKCδ-TRPA1 can lead to impaired calcium homeostasis in neurons, subsequently inducing ER stress. Western blot results revealed that the expression levels of GRP78, CHOP, and Caspase-12 were significantly upregulated following SCI. However, EA treatment stabilized calcium channels and consequently reduced the expression of GRP78, CHOP, and Caspase-12 (Figure 3e and i–k). These findings suggest that EA treatment mitigates ER stress induced by SCI. According to the results of the tissue immunofluorescence assay, the colocalization of NeuN and CHOP was significantly reduced following EA treatment (Figure 3b and d), providing further evidence of the role of EA in mitigating ER stress.

### Electroacupuncture treatment inhibits neuronal apoptosis in mice after spinal cord injury

**Figure 4:**
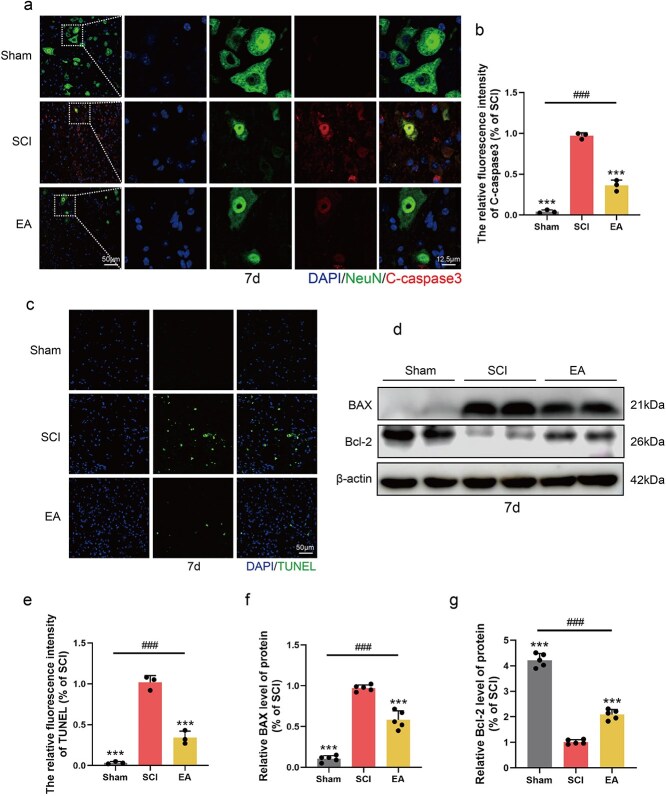
EA treatment inhibited neuronal apoptosis in SCI mice. (**a**) Immunofluorescence staining showed the expression of DAPI (blue), NeuN (green), and C-caspase3 (red) in each group 7 days post-SCI. Scale bar: 50 μm; 12.5 μm. (**b**) Statistical analysis of fluorescence intensity of C-caspase3 in each group, and the data are represented as mean ± SD (*n =* 3). (**c**) TUNEL staining in each group 7 days post-SCI. Scale bar: 50 μm. (**d**) Western blotting showing the expression of BAX, Bcl-2 in each group. (**e**) Statistical analysis of fluorescence intensity of TUNEL in each group, and the data are represented as mean ± SD (*n =* 3). (**f**, **g**) Quantitative analysis of protein expression in each group and the data are represented as mean ± SD (*n =* 5). ^***^ represents *P* < 0.001. ^###^ represents the sham group *vs* the EA group, *P* < 0.001. *EA* electroacupuncture, *SCI* spinal cord injury.

Neuronal survival is intricately linked to the treatment outcomes of SCI. Following our confirmation that EA can reduce inflammation levels in the injured area and inhibit ER stress via the PKCδ-TRPA1 pathway, we further investigated whether EA can promote neuronal survival. Immunofluorescence analysis of tissue labelled with NeuN and C-caspase3 revealed that high-intensity colocalization of NeuN and C-caspase3 occurred following SCI. However, after EA treatment, the intensity of colocalization between NeuN and C-caspase3 was significantly reduced, whereas the number of surviving neurons in the injured area increased (Figure 4a and b). These findings indicate that EA effectively reduces neuronal apoptosis and promotes nerve repair. TdT-mediated dUTP Nick-End Labeling (TUNEL) staining revealed that EA treatment also reduced the number of TUNEL-positive puncta in the injured area, further demonstrating the protective effect of EA on neurons (Figure 4c and e). The western blot results revealed that compared with those in the sham group, the levels of the pro-apoptotic proteins Bax were significantly greater in the SCI group. EA treatment not only reduced the levels of proapoptotic proteins but also upregulated the expression of the antiapoptotic protein Bcl-2, thereby alleviating apoptosis following SCI (Figure 4d, f, g). In conclusion, EA treatment effectively inhibited neuronal apoptosis in mice after SCI.

### Electroacupuncture promotes neurogenesis and axonal regeneration after spinal cord injury by promoting the secretion of neurotrophic factors

**Figure 5:**
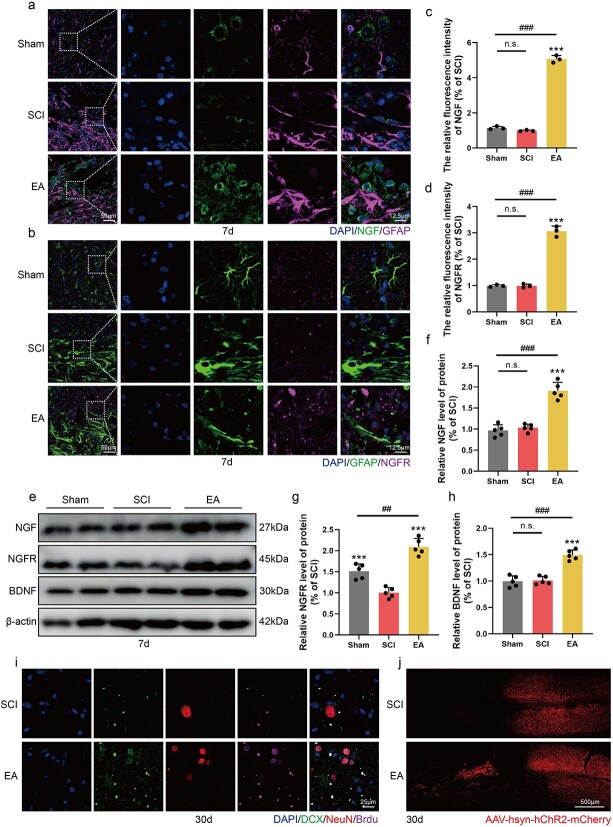
EA treatment promotes nerve and axon repair after SCI by promoting the secretion of neurotrophic factors. (**a**) Immunofluorescence staining showed the expression of DAPI (blue), NGF (green), and GFAP (pink) in all groups 7 days post-SCI. Scale bar: 50 μm; 12.5 μm. (**b**) Immunofluorescence staining showed the expression of DAPI (blue), GFAP (green), and NGFR (pink) in all groups 7 days post-SCI. Scale bar: 50 μm; 12.5 μm. (**c**, **d**) Statistical analysis of fluorescence intensity of NGF, NGFR in each group, and the data are represented as mean ± SD (*n =* 3). (**e**) Western blotting showing the expression of NGF, NGFR, and BDNF in each group. (**f**–**h**) Quantitative analysis of protein expression in each group and the data are represented as mean ± SD (*n =* 5). (**i**) Immunofluorescence staining showed the expression of DAPI (blue), DCX (green), NeuN (red), and BrdU (pink) in all groups 30 days post-SCI. Scale bar: 25 μm. (**j**) CST tracing in each group 30 days post-SCI. Scale bar: 500 μm. ^***^represents *P* < 0.001. ^##^, ^###^ represent sham group *vs* EA group respectively, *P* < 0.01., *P* < 0.001. n.s. represents no statistical significance. *EA* electroacupuncture, *SCI* spinal cord injury.

Neuronal repair involves a complex interplay of multiple factors, with neurotrophic factors playing crucial roles in neuronal differentiation, repair, and axonal regeneration. EA can upregulate the expression of neurotrophic factor–related genes, such as those involved in the synthesis and release of NGF and BDNF, thereby promoting neuronal and axonal repair following SCI. In tissue immunofluorescence experiments conducted 7 days post-EA treatment, we used GFAP to delineate the lesion border and costaining with NGF and NGFR to investigate the EA-induced promotion of NGF secretion. EA treatment significantly increased NGF and NGFR expression in the injured area compared with that in both the sham group and the SCI group, suggesting that EA facilitates neuronal repair by increasing NGF secretion (Figure 5a–d). NGF, NGFR, and BDNF protein levels were quantified by western blot analysis. Compared with both the Sham and SCI groups, the EA treatment resulted in a significant increase in the levels of these three proteins (Figure 5e–h). On the basis that EA promotes the secretion of neurotrophic factors, we verified the effect of EA on neurogenesis by costaining for DCX/NeuN/BrdU. The data revealed that NeuN-positive puncta appeared at the injury boundaries in the SCI group, but no colocalization with DCX or BrdU was observed; these neurons may represent residual populations after injury. In the EA group, we detected the colocalization of DCX/NeuN/BrdU at the injury boundary, indicating that EA effectively promotes neurogenesis (Figure 5i). Furthermore, we introduced CST tracing technology to monitor axonal regeneration. Almost no mCherry-positive points were detected in the downstream area of the SCI group. However, in the EA group, we detected many mCherry-positive points in the downstream area, indicating that EA can effectively promote the reconnection of downstream neural circuits (Figure 5j). In conclusion, EA promotes the secretion of neurotrophic factors to achieve neurogenesis and axonal regeneration in SCI mice.

### Electroacupuncture promotes nerve repair after spinal cord injury

**Figure 6:**
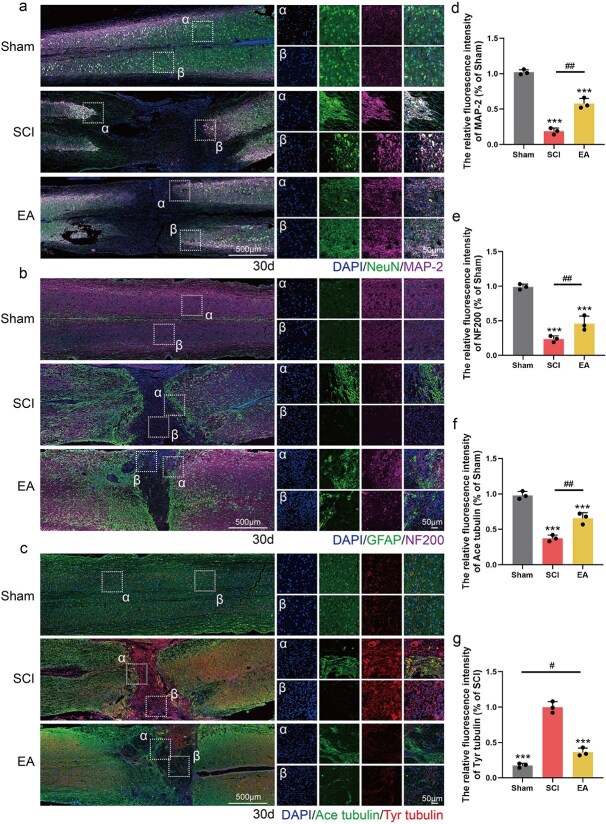
EA treatment promoted nerve and axon regeneration in the injured area, and promoted axonal repair by improving microtubule stability. (**a**) Immunofluorescence staining showed the expression of DAPI (blue), NeuN (green), and MAP-2 (pink) in all groups 30 days post-SCI. Scale bar: 500 μm; 50 μm. (**b**) Immunofluorescence staining showed the expression of DAPI (blue), GFAP (green), and NF200 (pink) in all groups 30 days post-SCI. Scale bar: 500 μm; 50 μm. (**c**) Immunofluorescence staining showed the expression of DAPI (blue), Ace tubulin (green), and Tyr tubulin (red) in all groups 30 days post-SCI. Scale bar: 500 μm; 50 μm. (**d**–**g**) Statistical analysis of fluorescence intensity of MAP-2, NF200, Ace tubulin, Tyr tubulin in each group, and the data are represented as mean ± SD (*n =* 3). ^***^ represents *P*<0.001. ^#^, ^##^ represent SCI group *vs* EA group or sham group *vs* EA group, respectively, *P* < 0.05, *P* < 0.01. *EA* electroacupuncture, *SCI* spinal cord injury.

**Figure 7:**
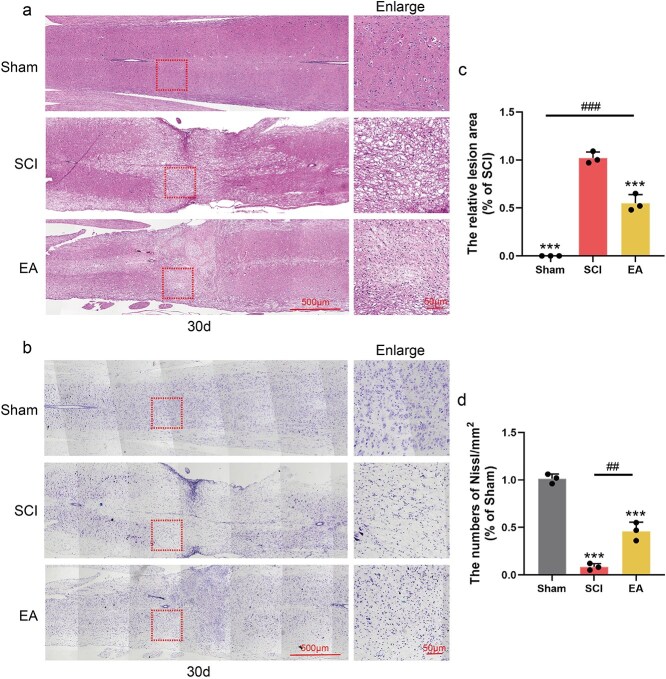
EA treatment promotes nerve repair after SCI. (**a**) Histomorphological images of HE stained in the Sham, SCI, EA group 30 days post-SCI. Scale bar: 500 μm; 50 μm. (**b**) Hiphographic images of Nissl staining in each group 30 days post-SCI. Scale bar: 500 μm; 50 μm. (**c**) Statistical analysis of the area of the injured area in each group. (**d**) Statistical analysis of the number of Nissl bodies in each group. ^***^ represents *P* < 0.001. ^##^ and ^###^ represent Sham group *vs* EA group or SCI group *vs* EA group, respectively, *P* < 0.01, *P* < 0.001. *EA* electroacupuncture, *SCI* spinal cord injury.

To further verify the nerve repair effect of EA in SCI mice, panoramic tissue immunofluorescence staining for NeuN and MAP-2 was performed 30 days after EA treatment. The results demonstrated that, compared with that in the SCI group, the SCI area in the SCI group was significantly reduced following EA treatment. These results suggest that compared with the control treatment, EA had a notable regenerative effect on neurons and dendrites within the injured region (Figure 6a and d). The proliferation of glial scars after SCI can block the extension of neurofilaments to the injury centre, and the extension of neurofilaments in the injury centre is important for the signal transduction of motor function. We used GFAP to label the glial scar and NF200 to label neurofilaments by tissue immunofluorescence staining to verify whether EA could facilitate the crossing of neurofilaments into the glial scar and entry into the injured region. Compared with those in the Sham group, the neurofilaments in the SCI group exhibited fragmentation and atrophy, with a reduction in fluorescence intensity. Additionally, GFAP staining revealed an enlargement of the injured area. Compared with those in the SCI group, a significant increase in NF200-positive spots was observed at the boundary of the lesion in the EA group, and NF200-positive spots were also detected within the lesion centre. These findings suggest that EA facilitates the passage of neurofilaments through the glial scar into the lesion core. Additionally, GFAP staining revealed a reduction in the size of the lesion area (Figure 6b and e). Axonal stability is crucial for axon regeneration and functional integrity. Ace-tubulin was used to label stable polymerized microtubules, while Tyr-tubulin was used to label unstable depolymerized microtubules. The results demonstrated that a broad range of Tyr-tubulin-positive areas emerged in the injured region following SCI, whereas the fluorescence intensity of Ace-tubulin was markedly suppressed. Following EA treatment, the fluorescence intensity of Tyr-tubulin significantly decreased, and the fluorescence intensity of Ace-tubulin notably increased (Figure 6c, f, and g). These findings indicate that EA treatment can increase microtubule stability, thereby maintaining axonal regeneration. H&E staining revealed that EA treatment improved the damage caused by SCI to tissue structure and reduced the injury area (Figure 7a and c). Nissl staining revealed that more Nissl bodies were present in the EA group than in the SCI group, indicating increased neuronal repair (Figure 7b and d). In conclusion, EA can promote nerve and axonal regeneration in the injured area and promote axonal repair by improving microtubule stability.

### TRPA1 expression plays an important role in neuronal apoptosis caused by endoplasmic reticulum stress after spinal cord injury

**Figure 8:**
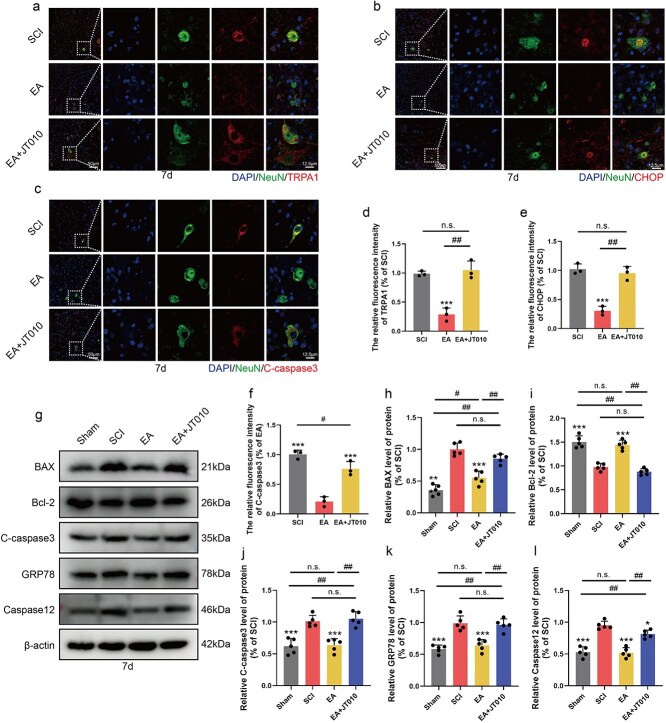
TRPA1 is a key pathway leading to endoplasmic reticulum stress–induced apoptosis in neurons after SCI. (**a**) Immunofluorescence staining showed the expression of DAPI (blue), NeuN (green), and TRPA1 (red) in all groups 7 days post-SCI. Scale bar: 500 μm; 50 μm. (**b**) Immunofluorescence staining showed the expression of DAPI (blue), NeuN (green), and CHOP (red) in all groups 7 days post-SCI. (**c**) Immunofluorescence staining showed the expression of DAPI (blue), NeuN (green), and C-caspase (red) in all groups 7 days post-SCI. Scale bar: 500 μm; 50 μm. (**d**–**f**) Statistical analysis of fluorescence intensity of TRPA1, CHOP, and C-caspase3 in each group, and the data are represented as mean ± SD (*n =* 3). (**g**) Western blotting showing the expression of BAX, Bcl-2, C-caspase3, GRP78, and Caspase12 in each group. (**h**–**l**) Quantitative analysis of protein expression in each group and the data are represented as mean ± SD (*n =* 5). ^**^ represents *P* < 0.01, ^***^ represents *P* < 0.001. ^#^ and ^##^ represent *P* < 0.05, *P* < 0.01. n.s. represents no statistical significance. *EA* electroacupuncture, *SCI* spinal cord injury.

To further investigate the role of TRPA1 in regulating ER stress, we administered the TRPA1 agonist JT010 concurrently with EA in SCI mice to determine whether TRPA1 activation mitigates the inhibitory effect of EA on ER stress. In the tissue immunofluorescence experiment conducted 7 days post-intervention, we costained samples with NeuN and TRPA1. Compared with that in the EA group, the TRPA1 fluorescence intensity in the EA + JT010 group was significantly greater (Figure 8a and d). Compared with that in the EA group, the fluorescence intensity of CHOP in the EA + JT010 group was significantly greater, suggesting that TRPA1 activation plays a crucial role in inducing ER stress in neurons (Figure 8b and e). Compared with that in the EA group, the colocalization intensity of NeuN and C-caspase3 in the EA + JT010 group was significantly greater, indicating that the neurons were undergoing apoptosis (Figure 8c and f). These findings suggest that TRPA1 activation may reverse the therapeutic effects of EA and induce neuronal apoptosis through ER stress. Western blot results revealed that ER stress–related proteins were significantly upregulated in the EA + JT010 group compared with those in the EA group, suggesting that a potential cascade effect initiated by TRPA1 activation subsequently led to ER stress. Moreover, the expression of the apoptosis-promoting proteins BAX and C-caspase3 significantly increased in response to JT010 treatment, whereas the expression of the apoptosis-inhibiting protein Bcl-2 decreased (Figure 8g–l). These findings indicate that TRPA1 disrupts the therapeutic effect of EA on neurons via the ER stress pathway. In conclusion, we further demonstrated that inhibition of ER stress by EA treatment reduced neuronal apoptosis by inhibiting TRPA1 activity.

## Discussion

In this study, we demonstrated that EA stimulation of the Shen Shu point attenuated excessive calcium influx, mitigated ER stress, decreased neuronal apoptosis, and facilitated nerve repair and motor function recovery following SCI. Based on these findings, we further observed that EA mitigated the inflammatory microenvironment following SCI, inhibited the activity of the PKCδ-TRPA1 pathway, and alleviated neuronal Ca^2+^ overload. Additionally, EA stimulation facilitated the secretion of neurotrophic factors, thereby promoting neuronal protection and regeneration.

In contemporary SCI treatment, surgical decompression and pharmacological interventions, such as methylprednisolone administration, are predominantly employed [35]. These approaches prioritize the immediate requirement to stabilize the patient’s condition; however, they fail to address chronic secondary injuries [36]. Secondary injury refers to the process that is gradually aggravated by local pathophysiological reactions on the basis of primary mechanical injury, including oxidative stress, the inflammatory response, and calcium homeostasis disorders. Together, these injuries aggravate neuronal apoptosis and hinder functional recovery. Among these factors, calcium overload and ER stress are key to neuronal degeneration following SCI [37].

Following SCI, mechanical damage directly compromises the integrity of neuronal cell membranes and the ER, leading to a significant influx of extracellular Ca^2+^ through multiple calcium channels [38]. Concurrently, oxidative stress or mechanical injury-induced calcium leakage from ER stores exacerbates cytosolic calcium overload [39]. It subsequently activates the calpain kinase, cleaves the pro-apoptotic protein Bax, and initiates the caspase-mediated apoptotic cascade. As ER stress intensifies, the antiapoptotic protein Bcl-2 is inhibited by the protein CHOP. Concurrently, ER-specific Caspase-12 is activated, ultimately leading to neuronal cell death [40]. TRPA1 is a calcium-permeable ion channel protein that is activated by Reactive Oxygen Species (ROS), proinflammatory cytokines, and mechanical stimuli. Activation of TRPA1 channels initiates a cascade of adverse effects, including the amplification of pain signals and inhibition of myelin formation [41]. Research has demonstrated that TRPA1 channel activation contributes to the development of peripheral diabetic neuropathy (PDN) and is mitigated by TRPA1 inhibitors [42]. Furthermore, TRPA1 activation leads to tau hyperphosphorylation and neuronal loss through ER stress, indicating its neurotoxic potential [43]. However, the precise mechanisms underlying its role in SCI remain to be elucidated. In conjunction with the effect of TRPA1 upregulation on calcium influx, our findings demonstrate that TRPA1 is activated by mechanical trauma and the inflammatory microenvironment following SCI. This activation leads to elevated expression of P-camkk2 and colocalization of CHOP and C-caspase3 in neurons. Western blot analysis further revealed increased expression levels of CHOP, caspase-12, and the apoptosis-related protein BAX, indicating that TRPA1 mediates neuronal apoptosis post-SCI. Several TRPA1 antagonists have been developed for treatment following TRPA1 activation [44]. However, our research demonstrated that TRPA1 is also regulated by upstream kinases. The expression of PKCδ, a member of the protein kinase C family, is activated in response to oxidative and inflammatory stimuli, thereby exacerbating downstream signalling cascades and leading to increased cellular stress. Studies have shown that in nonalcoholic fatty liver disease, PKCδ activation promotes lipid accumulation and ER stress by upregulating the unfolded protein response markers Bip and xbp-1. In diabetic neuropathy, PKCδ activation is associated with mitochondrial dysfunction and neuronal apoptosis [45, 46]. Following SCI, TRPA1 is activated, leading to the opening of calcium channels. Although activated PKCδ does not directly interact with calcium channels, it phosphorylates TRPA1, thereby potentiating its Ca^2+^ channel activity and conductance [47]. The resulting increase in the intracellular Ca^2+^ concentration subsequently activates PKCδ, establishing a positive feedback loop that culminates in mitochondrial dysfunction, ROS overproduction, and exacerbated ER stress. Consequently, our study focused on the microenvironment and calcium homeostasis maintenance following SCI. Prior research has demonstrated that TRPA1 antagonists, such as HC-030031, can alleviate pain; however, they do not address the issue of upstream kinase activation [48]. In contrast, the dual inhibition of PKCδ and TRPA1 off ER stress is a comprehensive strategy for maintaining neuronal Ca^2+^ homeostasis following SCI through the targeting of the PKCδ-TRPA1 pathway.

EA is a therapeutic modality that combines traditional acupuncture with contemporary low-frequency electrical stimulation. By delivering controlled low-frequency currents to acupuncture points, EA elicits both mechanical and electrophysiological responses. EA has been extensively utilized as a nonpharmacological intervention for various types of neurological disorder stress. EA affects multiple neurological conditions, including chronic stress–induced depression, where it modulates the JNK and mTOR pathways to enhance synaptic plasticity [49]. In patients with poststroke hemiplegia, EA aids in the recovery of motor and sensory functions [50]. Additionally, EA mitigates neuroinflammation in ischaemic stroke; however, the precise molecular mechanisms underlying its efficacy in treating SCI remain to be elucidated [27]. Compared with traditional acupuncture, which modulates inflammation via mechanical stimulation, EA may offer superior efficacy in regulating inflammatory factors through the addition of electrical stimulation. EA significantly suppresses the expression of proinflammatory cytokines such as IL-6 and TNF-α while upregulating the expression of the anti-inflammatory mediator IL-10 by activating specific neuroendocrine pathways. This includes the inhibition of key inflammatory signalling pathways, such as the TLR4/NF-κB pathway [51]. Consequently, EA plays a crucial role in mitigating neuroinflammation and protecting the microenvironment in cases of chronic secondary injury following SCI. In our study, we observed that stimulation with EA at the Shen Shu point led to a reduction in the expression of the M1-type microglial marker CD86 and upregulation of the M2-type microglial markers Arg1 and CD206. These findings indicate that EA treatment has a therapeutic effect on the microenvironment following SCI. We subsequently discovered that mitigating the inflammatory milieu inhibits PKCδ-TRPA1 pathway activation, thereby regulating neuronal calcium overload mediated by TRPA1 channel activation through PKCδ. This process helps reduce neuronal apoptosis induced by ER stress and prevents sustained neuronal apoptosis in SCI. To further elucidate the role of TRPA1 in neuronal calcium regulation, we administered the TRPA1 agonist JT010 *in vivo*. Our findings revealed that the expression levels of ER stress–related proteins, including Caspase12 and GRP78, were significantly upregulated in the EA + JT010 group. Additionally, the levels of apoptosis-related proteins markedly increased. Moreover, a large number of neurons were positively colocalized with CHOP. These results indicate that JT010 treatment enhances the expression of ER stress– and apoptosis-related proteins in neurons, suggesting that TRPA1 overexpression may induce ER stress and apoptosis, thereby potentially compromising the therapeutic efficacy of EA.

Moreover, EA induces the secretion of neurotrophic factors, including BDNF and NGF, from both neurons and glial cells. This represents an additional critical mechanism contributing to neuronal repair following EA treatment. Several studies have demonstrated that NGF and BDNF inhibit neuronal apoptosis following SCI and protect neuronal perinuclear bodies from secondary damage [28, 52]. Additionally, EA increases microtubule polymerization, thereby providing structural support for axonal extension [53]. In our study, following EA treatment, a significant number of NGF- and NGFR-positive sites were observed in both the injured area and its periphery in SCI patients, with a concurrent increase in their protein levels. Further histological analysis revealed colocalization of DCX/NeuN/BrdU at the injury margin. In CST tracing, we also observed a large number of mCherry-positive spots downstream of the injury, demonstrating that the neurotrophic factor secretion effect of EA has a positive effect on neurogenesis and axonal regeneration. On the other hand, EA regulates tubulin expression by decreasing the level of Tyr-tubulin and increasing the level of Ace-tubulin. These findings indicate that EA promotes axonal elongation through a mechanism that stabilizes microtubules. In the subsequent evaluation of mouse kinematics, mice treated with EA achieved higher BMS scores, exhibited superior electrical signal transduction capabilities, and obtained better hindlimb motor function scores.

The present study is the first to elucidate the deleterious effect of the PKCδ-TRPA1 axis on ER stress caused by Ca^2+^ overload in secondary-stage SCI neurons. Furthermore, EA stimulation of the Shen Shu acupoint has been shown to protect neurons by inhibiting this pathway and promoting neurogenesis and microtubule stability through the stimulation of neurotrophic factor secretion, thereby enhancing motor function recovery in SCI mice. On the basis of these findings, we aim to offer novel insights into the relationship between the neuroprotective effects of EA and the molecular pathways involved in SCI pathology.

## Conclusions

In summary, on the basis of the pathological phenomenon of neuronal Ca^2+^ overload after SCI and the intervention methods of EA, we found the following: (i) mechanical injury and the inflammatory microenvironment after SCI activate PKCδ-TRPA1, which is important for neuronal ER stress and apoptosis caused by neuronal calcium overload. (ii) EA treatment reduces PKCδ-TRPA1 expression as well as the ER stress and apoptosis caused by neuronal calcium overload. (iii) EA facilitated neurogenesis by promoting the secretion of neurotrophic factors and promoted axon elongation by stabilizing microtubules, thus promoting the recovery of motor function in SCI mice.

## Data Availability

All data are available in the main text or the supplementary materials.
